# Inflammatory Biomarkers in Refractory Congestive Heart Failure Patients Treated with Peritoneal Dialysis

**DOI:** 10.1155/2015/590851

**Published:** 2015-10-11

**Authors:** Margarita Kunin, Vered Carmon, Michael Arad, Nomy Levin-Iaina, Dov Freimark, Eli J. Holtzman, Dganit Dinour

**Affiliations:** ^1^Nephrology and Hypertension Institute, Sheba Medical Center and Sackler Faculty of Medicine, 5265601 Tel-Hashomer, Israel; ^2^Heart Failure Service and Heart Institute, Sheba Medical Center and Sackler Faculty of Medicine, 5265601 Tel-Hashomer, Israel

## Abstract

Proinflammatory cytokines play a pathogenic role in congestive heart failure. In this study, the effect of peritoneal dialysis treatment on inflammatory cytokines levels in refractory congestive heart failure patients was investigated. During the treatment, the patients reached a well-tolerated edema-free state and demonstrated significant improvement in NYHA functional class. Brain natriuretic peptide decreased significantly after 3 months of treatment and remained stable at 6 months. C-reactive protein, a plasma marker of inflammation, decreased significantly following the treatment. Circulating inflammatory cytokines TNF-*α* and IL-6 decreased significantly after 3 months of peritoneal dialysis treatment and remained low at 6 months. The reduction in circulating inflammatory cytokines levels may be partly responsible for the efficacy of peritoneal dialysis for refractory congestive heart failure.

## 1. Introduction

Removal of extensive fluid overload is one of the most difficult challenges in the management of severe congestive heart failure (CHF), particularly in patients who are refractory to diuretic therapy. Peritoneal ultrafiltration (UF) is a simple choice for daily fluid removal. Today, peritoneal dialysis (PD) is increasingly used to treat hypervolemic CHF patients who are resistant to conventional therapies, in particular when complicated by renal insufficiency (reviewed in [1, 2]). It was demonstrated that PD improves the functional status, reduces hospitalization rate, and even may decrease mortality rate [3–6].

The link between HF and inflammation was recognized and reported in 1990 by Levine et al. [7], who noted that levels of an inflammatory cytokine, tumor necrosis factor (TNF), were elevated in the setting of HF. Since this report, a number of studies have shown that, in addition to TNF, other proinflammatory cytokines and chemokines are also involved in cardiac function depression and progression of HF (reviewed in [8, 9]). It has been identified that biologically active molecules such as the cytokines are expressed in the setting of heart failure [10–13]. In many forms of cardiomyopathic left ventricular (LV) dysfunction, there is a rapid myocardial expression of proinflammatory cytokines such as interleukin-1 (IL-1), interleukin-8 (IL-8), and tumor necrosis factor-alpha (TNF-*α*), which mediate, via specific receptors, various processes as gene expression, cell growth, or apoptosis [14–16].

Myocardial expression of cytokines contributes to depression of contractile performance and adverse LV remodelling. Cytokine-induced decreased contractile performance appears to result from sphingosine production, which interferes with myocardial calcium handling [15]. The activity of inflammatory cytokines is also influenced by anti-inflammatory cytokines such as transforming growth factor (TGF-*β*) and interleukin-10 (IL-10), which can downregulate the production of several inflammatory cytokines from macrophages and other cells [17, 18]. Peripheral-circulating levels of these cytokines are elevated in patients with heart failure and correlate with disease severity [8]. Several studies have shown that haemodiafiltration (HDF) using porous synthetic membranes removes a wide range of circulating inflammation mediators [19–22] and can also influence circulating plasma concentrations of various mediators such as cytokines.

While emerging as an effective treatment option for refractory heart failure, peritoneal dialysis may by itself contribute to systemic inflammation (reviewed in [23]). The continuous presence of dialysis fluid with a high glucose concentration and glucose degradation products (GDPs), prolonged exposure to conventional bioincompatible glucose-based PD solutions, loss of residual renal function, and increased body fat mass all contribute to systemic inflammation in PD patients [23].

This study was designed to evaluate the net effect of peritoneal dialysis on circulating inflammatory and anti-inflammatory cytokine levels in patients with refractory CHF and fluid overload.

## 2. Subjects and Methods

### 2.1. Subjects

Patients with refractory CHF who were referred by their cardiologists to our PD unit between March 2012 and July 2014 and completed at least 3-month period of follow-up were enrolled into this study. The study protocol was approved by the Sheba Medical Center Institutional Human Research Board. All patients were in NYHA functional class IIIb or IV and showed symptoms and signs of severe cardiac failure with volume excess. They were receiving maximal therapy according to the heart failure guidelines including dietary fluid and salt restriction and maximal tolerable drug treatment, including diuretics, loop and distal tubule (metolazone), angiotensin-converting enzyme inhibitor (ACE-I) or angiotensin II receptor blockers (ARB), beta-blockers, and digoxin. Some patients were also treated with intravenous furosemide and vasoactive agents in a CHF day care center. The inclusion criteria for PD were all of the following: (1) NYHA functional class IIIb or IV; (2) an echocardiographic evidence of significant left or right ventricular dysfunction, valvular heart disease, or pulmonary hypertension; (3) significant volume overload despite maximal doses of diuretics or repeated episodes of deteriorating kidney function (defined as a 50% increase in serum creatinine from basal concentration) during the intensification of diuretics treatment or recurrent hospitalization for volume overload in the preceding 3 months. Patients with contraindication for PD (such as severe lung disease, extensive abdominal scars, and abdominal aortic aneurism) or those incapable of learning and complying with the procedure of PD were excluded from the study. PD catheter was implanted by surgical dissection under local anesthesia in the operating room. In patients with ascites, peritoneal centesis was started by a specially trained PD nurse a day after Tenckhoff catheter insertion. In patients with significant volume overload, small volume exchanges (around 1,500 mL) were performed in the recumbent position by dialysis nurse starting the day after catheter placement. Until the patient and/or family member have learnt the dialysis technique (which usually takes 2-3 weeks), UF was performed by a PD nurse in the PD unit every day or every other day.

### 2.2. Clinical Evaluation

The following clinical parameters were collected: disease etiology and functional status (NYHA), preserved/reduced LV function, comorbidities and medications, body weight, and mean arterial blood pressure. Assessment of fluid status was based on clinical examination. Laboratory investigations included serum hemoglobin and leukocyte count, serum albumin, sodium, urea, creatinine, uric acid, erythrocyte sedimentation rate (ESR), and C-reactive protein (CRP). Primary kidney disease was defined as urine protein >0.5 g/24 h, abnormal urine microscopy, and/or abnormal renal sonography (e.g., unequal kidney sizes or reduced kidney parenchyma). Patients with urine protein <0.5 g/24 h, normal urine microscopy, and normal kidneys per sonography were classified as having cardiorenal syndrome. Echocardiographic parameters used in the study included LVEF and RVEF and systolic pulmonary artery pressure (SPAP). Glucose- and non-glucose-containing (icodextrin) dialysis solutions (Teva Medical, Israel, and Cure Medical and Technical Supply, Fresenius, Germany) were used in the study. Data on the type and volume of PD solutions used by the patient and daily peritoneal UF volume were gathered.

### 2.3. Cytokine ELISA Assays

Plasma levels of TNF-*α*, IL-6, and IL-10 were assessed by enzyme-linked immunosorbent assay (ELISA) according to supplier protocols (R&D systems). This assay employed the quantitative sandwich enzyme immunoassay technique. The cut-off or lower limit of sensitivity was 0.106 pg/mL for TNF-*α*, 0.039 pg/mL for IL-6, and 0.09 pg/mL for IL-10.

### 2.4. BNP Assay

BNP was measured using the Alere Triage BNP Test, a rapid fluorescence immunoassay kit.

### 2.5. Statistical Analysis

**Figure 1:**
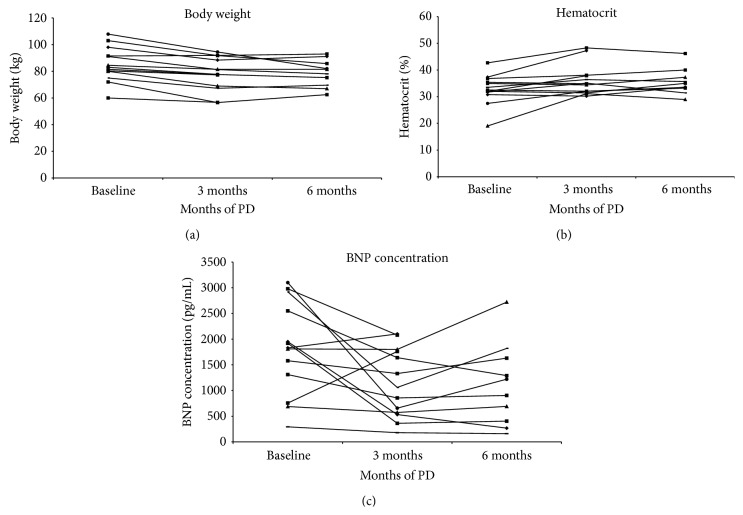
Selected clinical and biochemical variables of patients with refractory CHF treated with PD. Individual patient trajectories are shown. *n* = 13. (a) Changes in body weight. (b) Changes in blood hematocrit. (c) Changes in circulating BNP levels.

**Figure 2:**
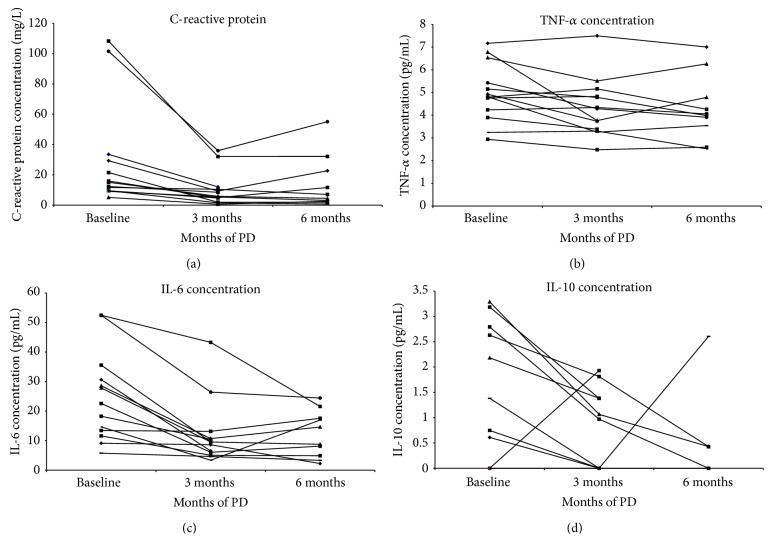
Circulating cytokine and C-reactive protein levels in patients with refractory CHF treated with PD. Individual patient trajectories are shown. *n* = 13. (a) Changes in serum C-reactive protein. (b) Changes in circulating TNF-*α* levels. (c) Changes in circulating IL-6 levels. (d) Changes in circulating IL-10 levels.

The data are presented as median and range for continuous variables and as absolute numbers and percentages for categorical variables. Data presented in Table 2 were compared by two-tailed Student's *t*-test. Data in Figures 1 and 2 were compared by two-tailed paired Student's *t*-test. Differences were considered significant for *P* < 0.05.

## 3. Results

### 3.1. Clinical Outcome

The clinical, biochemical, and echocardiographic characteristics of the patients at baseline, prior to beginning of PD, are presented in Table 1. During the follow-up period, 3 patients died. Two died from CHF exacerbation and one diabetic patient died from septic foot complications. All patients continued treatment with oral furosemide. The median dose of oral furosemide the patients received did not change during follow-up: it was 160 mg per day (range 120–240 mg). Four patients were treated with metolazone regularly; another 4 were instructed to add metolazone when their body weight went up. The usual dose of metolazone was 2.5 mg twice a week. Patients who were treated in CHF day care center intravenous furosemide and vasoactive agents (8 out of 13 patients) continued the treatment while on PD.

The median amount of PD solutions used per patient was 2 liters per day (range 2–12 liters). All patients reached a well-tolerated edema-free state during the first months after starting PD. By the end of the first 3 months of treatment, body weight of CHF patients decreased significantly due to fluid loss (Figure 1(a)). The median weight loss was 5.3 kg (*P* = 0.0001). Table 2 presented selected clinical and biochemical characteristics during patients' follow-up. The clinical benefit of PD manifested by improved NYHA functional class by a median of one class, from NYHA IV to III, which remained stable at 6 months of treatment (*P* = 0.0035). Blood hematocrit increased significantly during the treatment from median of 32.51 (19.03–42.68) to 34.84 (30.19–48.28) at 3 months (*P* = 0.0156) and to 34.29 (28.94–46.19) at 6 months (*P* = 0.0051, Figure 1(b)).

### 3.2. Circulating BNP Levels

Elevated pretreatment circulating BNP levels were found in all patients (Figure 1(c)). BNP levels decreased significantly from a median of 1830 (294–3100) to 1060 (180–2100) pg/mL (*P* = 0.0259) at 3 months and measured 1062 (161–2720) pg/mL (*P* = 0.0385) at 6 months.

### 3.3. C-Reactive Protein and Circulating Cytokine Levels

Baseline C-reactive protein (Figure 2(a)) was approximately 6-fold above the upper level of normal. There was a substantial drop in serum C-reactive protein concentration during the treatment. Median serum CRP decreased from 15.07 (5.09–108.3) at baseline to 5.81 (0.63–35.94) mg/L (*P* = 0.0139) at 3 months and was 5.78 (0.74–55.09) mg/L (*P* = 0.0375) after 6 months of PD.

Circulating TNF-*α* level (Figure 2(b)) decreased significantly from 4.81 (2.94–7.17) pg/mL at baseline to 4.29 (2.48–7.5) pg/mL (*P* = 0.0313) at 3 months and measured 4.02 (2.52–7.01) pg/mL (*P* = 0.0028) after 6 months of PD.

Circulating level of IL-6 (Figure 2(c)) decreased from median of 22.57 (5.74–52.46) pg/mL at baseline to 9.53 (3.34–43.29) pg/mL (*P* = 0.0004) at 3 months and was 11.68 (2.22–24.43) pg/mL (*P* = 0.0133) after 6 months of PD.

Median serum anti-inflammatory cytokine IL-10 levels (Figure 2(d)) decreased from 0.75 (0–3.29) pg/mL at baseline to 0 (0–1.93) pg/mL (*P* = 0.056) at 3 months and remained undetectable (range 0–2.6) pg/mL (*P* = 0.0974) at 6 months.

### 3.4. Preserved LV and RV Function

Inside the subgroups of patients, it was found that circulating TNF-*α* and IL-6 decreased unsignificantly in patients with preserved LV function. Circulating TNF-*α* level decreased from median of 5.97 (3.9–7.17) pg/mL at baseline to 4.28 (3.38–7.5) pg/mL (*P* = 0.31; *n* = 4) at 3 months and to 4.78 (3.4–7.01) pg/mL (*P* = 0.1715; *n* = 4) after 6 months of PD in patients with preserved LV function compared to TNF-*α* drop from 4.79 (2.94–6.53) pg/mL at baseline to 4.29 (2.48–5.51) pg/mL (*P* = 0.055; *n* = 9) at 3 months and to 3.91 (2.52–6.26) pg/mL (*P* = 0.012; *n* = 9) after 6 months in patients with low LV function. Circulating level of IL-6 decreased from median of 15.83 (9.11–27.83) pg/mL at baseline to 9.98 (8.58–13.13) pg/mL (*P* = 0.2102; *n* = 4) at 3 months and to 8.75 (2.22–17.55) pg/mL (*P* = 0.3915; *n* = 4) after 6 months of PD in patients with preserved LV function compared to IL-6 drop from 28.55 (5.74–52.46) pg/mL at baseline to 6.49 (3.34–43.29) pg/mL (*P* = 0.0009; *n* = 9) at 3 months and to 14.6 (3.3–24.43) pg/mL (*P* = 0.029; *n* = 9) after 6 months in patients with low LV function. In patients with preserved LV function, the cause of CHF was nonischemic. Two patients had diastolic heart failure, one patient had restrictive cardiomyopathy with severe pulmonary hypertension of unknown cause, and one had primary pulmonary hypertension with right heart failure.

In patients with preserved RV function, circulating IL-6 levels decreased insignificantly following PD treatment from median of 13.4 (5.74–52.46) pg/mL at baseline to 8.58 (3.34–43.29) pg/mL (*P* = 0.1323; *n* = 5) at 3 months and was 16.95 (2.22–21.56) pg/mL (*P* = 0.3466; *n* = 5) after 6 months of PD in patients with preserved RV function compared to IL-6 fall from 28.19 (11.6–52.46) pg/mL at baseline to 9.56 (5.02–26.42) pg/mL (*P* = 0.0003; *n* = 8) at 3 months and to 8.75 (4.87–24.43) pg/mL (*P* = 0.0094; *n* = 8) after 6 months in patients with decreased RV function.

### 3.5. Treatment with ACE-I/ARBs and Spironolactone

Inside the subgroups of patients treated with ACE-I/ARBs or spironolactone, it was found that patients treated with ACE-I/ARBs demonstrated more significant decrease in inflammatory cytokines compared to the group without those drugs: TNF-*α* at 3 months and IL-6 at 3 and 6 months decreased significantly in treated group compared to significant decrease at 6 months for TNF-*α* and at 3 months for IL-6 in patients without those drugs. The differences between treated and nontreated groups were less consistent for spironolactone.

Circulating TNF-*α* level decreased from median of 4.93 (2.94–6.53) pg/mL at baseline to 4.29 (2.48–5.51) pg/mL (*P* = 0.0403; *n* = 5) at 3 months and to 3.98 (2.59–6.26) pg/mL (*P* = 0.1618; *n* = 5) after 6 months of PD in patients treated with ACE-I or ARBs compared to TNF-*α* drop from 4.8 (3.24–7.17) pg/mL at baseline to 4.28 (3.25–7.5) pg/mL (*P* = 0.2027; *n* = 8) at 3 months and to 4.12 (2.52–7.01) pg/mL (*P* = 0.014; *n* = 8) after 6 months in patients without ACE-I or ARBs. Circulating level of IL-6 decreased from median of 28.55 (11.6–52.46) pg/mL at baseline to 6.49 (5.02–26.42) pg/mL (*P* = 0.006; *n* = 5) at 3 months and to 11.35 (4.87–24.43) pg/mL (*P* = 0.038, *n* = 5) after 6 months of PD in patients treated with ACE-I or ARBs compared to IL-6 drop from 16.44 (5.74–52.46) pg/mL at baseline to 9.56 (3.34–43.29) pg/mL (*P* = 0.0241; *n* = 8) at 3 months and to 12.85 (2.22–21.56) pg/mL (*P* = 0.1745; *n* = 8) after 6 months in patients without ACE-I or ARBs.

Circulating TNF-*α* level decreased from median of 4.87 (2.94–5.43) pg/mL at baseline to 3.49 (2.48–4.29) pg/mL (*P* = 0.0177; *n* = 4) at 3 months and to 3.54 (2.59–3.91) pg/mL (*P* = 0.987; *n* = 4) after 6 months of PD in patients treated with spironolactone compared to TNF-*α* drop from 4.79 (3.24–7.17) pg/mL at baseline to 4.78 (3.3–7.5) pg/mL (*P* = 0.2457; *n* = 9) at 3 months and to 4.26 (2.52–7.01) pg/mL (*P* = 0.0289; *n* = 9) after 6 months in patients without spironolactone. Circulating level of IL-6 decreased from median of 26.61 (5.74–52.46) pg/mL at baseline to 6.26 (4.61–26.42) pg/mL (*P* = 0.0589; *n* = 4) at 3 months and to 8.1 (3.3–24.43) pg/mL (*P* = 0.1799; *n* = 4) after 6 months of PD in patients treated with spironolactone compared to IL-6 drop from 18.25 (9.11–52.46) pg/mL at baseline to 9.58 (3.34–43.29) pg/mL (*P* = 0.0051; *n* = 9) at 3 months and to 14.6 (2.22–21.56) pg/mL (*P* = 0.0716; *n* = 9) after 6 months in patients without spironolactone.

## 4. Discussion

Our study confirmed that PD treatment effectively removes fluid overload in patients with refractory CHF. Brain natriuretic peptide (BNP) levels measured 18-fold normal before the treatment, decreased significantly after 3 months of treatment, and remained stable at 6 months. Secretion of natriuretic peptides, BNP and amino-terminal pro-B-type natriuretic peptide (NTpro-BNP), is stimulated by ventricular stretch and wall tension in CHF. Both BNP and NT-pro-BNP plasma concentration have been shown to be useful in the diagnosis [24] and risk stratification [25, 26] of HF. It was demonstrated that conventional therapies for heart failure including diuretics, angiotensin-converting enzyme inhibitors/angiotensin receptor blockers, and *β*-blockers lower natriuretic peptide values [27]. Patients referred by cardiologists to PD in our study already received maximal tolerable drug treatment. It seems that peritoneal dialysis provided additional benefit and caused decrease of BNP levels in those patients.

CRP is liver-derived protein that is regulated by interleukin-6 [28]. CRP has been described to correlate with disease severity and prognosis in HF [29–33]. The role of CRP in the prediction of development of HF was also reported [34]. Use of ACE inhibitors and beta-blockers has been associated with lower levels of CRP in HF patients [35]. At the present time, despite its clear associations with HF disease severity and outcomes, it is not clear whether CRP is merely a marker of inflammation with no particular role in the development of HF or whether it is involved in the pathogenesis and progression of HF. CRP levels were elevated 6-fold upper normal limit in our patients. PD treatment led to significant decrease in CRP. Two other routine laboratory tests, erythrocyte sedimentation rate and white blood cell count, did not change significantly during the treatment.

Accumulating evidence indicates that proinflammatory cytokines play a pathogenic role in CHF. Inflammatory cytokines may modulate myocardial functions by a variety of mechanisms including stimulation of hypertrophy and fibrosis through direct effects on cardiomyocytes and fibroblasts, impairment of myocardial contractile function through direct effects on intracellular calcium transport, and signal transduction through *β*-adrenergic receptors, induction of apoptosis, and stimulation of genes involved in myocardial remodeling [8]. Inflammatory mediators could also contribute more indirectly to the progression of HF through impairment of bone marrow function with secondary anemia and inappropriate endothelial cell activation and impairment of peripheral muscle with secondary induction of systemic inflammation and reflex abnormalities in HF [8]. Peripheral-circulating as well as intracardiac levels of these cytokines are elevated in patients with HF [7, 10, 12, 13, 36]. TNF-*α* and IL-6 circulating levels are elevated and correlate with disease severity in heart failure (reviewed in [9]). Proinflammatory molecules are activated starting at earlier phases of HF asymptomatic left ventricular dysfunction and continue to rise in direct relation to worsening NYHA functional class regardless of the etiology of HF [9, 34]. Circulating levels of TNF, IL-6, and TNF soluble receptors (sTNFR1 and sTNFR2) have been reported to predict poorer survival [9]. Most studies have evaluated patients with HF and depressed ejection, but it was demonstrated that higher TNF levels were independently associated with a greater risk of mortality even in patients with HF and preserved ejection fraction [37].

Clinical studies have shown that treatment with angiotensin receptor antagonists can lead to significant reductions in circulating levels of TNF in patients with HF [38]. *β*-adrenergic blockade has also been shown to result in significant reductions in proinflammatory cytokine levels in clinical studies with HF patients [39–44]. Treatment with the long-acting dihydropyridine calcium antagonist, amlodipine, for a period of 26 weeks lowered plasma IL-6 levels in patients with HF [45]. Other studies have noted that optimization of background standard therapy of HF with diuretics, ACE inhibitors, beta-blockers, and digoxin can result in significant reductions in circulating levels of TNF and IL-6 [46]. Our findings indicate that peritoneal dialysis markedly reduced circulating proinflammatory cytokine TNF-*α* and IL-6 levels showing additional benefit to already maximally tolerate traditional drug regiments. The interesting finding was an insignificant effect of PD treatment on TNF and IL-6 levels in patients with preserved LV function and on IL-6 level in patients with preserved RV function. This result needs further confirmation on large sample size. Larger patient's group size is also needed to separate the effect of PD on inflammatory cytokines from the effects of such standard drugs as ACE-I, previously shown to reduce circulating proinflammatory cytokines [38].

The proinflammatory cytokine response is controlled by a series of immunoregulatory molecules, termed the “anti-inflammatory” cytokines. These cytokines act in concert with specific cytokine inhibitors and soluble cytokine receptors to regulate the human immune response. Their physiologic role in inflammation and pathologic role in HF are being increasingly recognized [47]. In several inflammatory disorders, the potential pathogenic effect of inflammatory cytokines will depend on the balance in the cytokine network, particularly on the levels of counteracting anti-inflammatory mediators. Patients with severe HF were found to have decreased levels of TGF-*β*1 and inadequately raised levels of IL-10 in relation to the elevated TNF concentrations, and these abnormalities in the cytokine network were most pronounced in patients with the most severe HF [48]. Although HF patients have enhanced expression of anti-inflammatory cytokine IL-10 compared to the normal population [39], in patients with severe HF, IL-10 levels in relation to the elevated TNF concentrations are considered inadequately raised [34]. Therefore, the balance is tipped toward enhanced expression of proinflammatory cytokines relative to anti-inflammatory cytokines in the HF population. IL-10 downregulates the production of inflammatory cytokines in a variety of cell types and enhances the release of sTNF receptors; thus, it is known that IL-10 has potential beneficial effects in terms of its cardioprotective properties in CHF [49, 50]. It was demonstrated that circulating levels of IL-10 increased in relation to elevated TNF-*α* levels in patients with dilated cardiomyopathy and may support the concept that the increase of IL-10 levels enhances the release of sTNFR2 [43]. Moreover, elevated levels of IL-10 were markedly decreased, in accordance with the reduction of TNF-*α* levels, due to beta-blocker therapy [43]. Therefore, IL-10 may be a potential therapeutic agent, as an immunoregulatory factor, in CHF [43]. We demonstrated that IL-10 levels also tended to decrease (albeit not significantly) during PD treatment and this decrease was accompanied by TNF-*α* reduction.

We do not think that removal of proinflammatory cytokines by peritoneal membrane had a significant impact on cytokine plasma levels. Most of our patients were treated with one or two dialysis exchanges per day; therefore, significant removal is unlikely. It was also demonstrated that clearances of high molecular weight compounds such as b2-microglobulin by PD are significantly lower as compared to the clearances of the uremic retention solutes urea nitrogen and creatinine because high molecular weight hampers their diffusive and convective transport through the pores of the peritoneal membrane [51]. It was proposed that UF in general cannot be expected to remove high molecular weight substances such as cytokines in clinically relevant amounts owing to its operative characteristics [52]. Neurohumoral activation reset towards a more physiological condition after fluid removal during PD treatment is probably responsible for proinflammatory cytokines reduction. It was proposed that there are important interactions between the renin-angiotensin, adrenergic systems, and proinflammatory cytokines and many of the conventional therapies for HF may work, at least in part through the modulation of proinflammatory cytokines.

We assume that PD treatment can lower the circulating level of proinflammatory TNF-*α* and IL-6 in patients with refractory CHF and fluid overload showing additional benefit to already maximally tolerated traditional drug regiments. The limitations of the present study include its small number of patients and the lack of control group. In this regard, we cannot rule out that the observed decrease in inflammatory biomarkers was due to regression to the mean, more careful clinical follow-up compared with routine standards of care, or Hawthorne's effect rather than PD for itself.

In this group of advanced CHF patients refractory to traditional drug therapy with extremely high BNP levels, the effect of PD treatment on circulating IL-6 was the most prominent finding. In this regard, IL-6 can serve as biomarker to guide therapy in those patients. It appears that CRP in which liver production is regulated by IL-6 could also be used as a reliable marker for therapy response, taking into account the fact that its role in pathogenesis and progression of HF is less clear. Large-scale trials are needed to check whether the changes in inflammatory biomarkers over time correlate with morbidity and mortality in HF patients.

## Figures and Tables

**Table 1:** Selected clinical, biochemical, and echocardiographic characteristics of the patients at baseline.

Age, years	64 (52–82)
Females	4 (31%)
Ischemic cardiomyopathy	8 (61%)
NYHA class III/IV	4/9
Diabetes mellitus	8 (62%)
History of hypertension	8 (62%)
Primary kidney disease	8 (62%)
Body weight, kg	83 (60–107.9)
Mean arterial blood pressure, mm Hg	85.3 (67–108.7)
LVEF, %	20 (7–60)
Preserved LV function	4 (31%)
RV dysfunction	8 (62%)
Estimated SPAP, mm Hg	56 (38–92)
CHF day care treatment	8 (62%)
Medications	
Loop diuretic	13 (100%)
Thiazide and thiazide-like diuretics, metolazone	4 (31%)
Spironolactone	4 (31%)
Beta-blockers	12 (92%)
Digoxin	6 (46%)
ACEI or ARB	5 (39%)

Values are expressed as median and range for continuous variables and as absolute numbers and percentages for categorical variables.

**Table 2:** Selected clinical and biochemical characteristics during patients' follow-up.

	Baseline	3 months	6 months	*P* value
NYHA class	4.0 (3.0–4.0)	3.0 (3.0–4.0)	3.0 (3.0–4.0)	0.0035
Serum creatinine, mg/dL	2.64 (1.54–5.89)	2.55 (1.36–6.27)	2.28 (1.32–8.11)	0.9907
Serum urea, mg/dL	210 (83–287)	135 (79–194)	143.5 (66–176)	0.0012
Serum sodium, mEq/L	135 (127–142)	134 (125–143)	138 (136–139)	0.1843
Serum uric acid, mg/dL	11.6 (4.5–14)	7.7 (6.3–13.7)	8.7 (7.3–11.6)	0.0585
Serum albumin, g/dL	3.2 (2.8–3.6)	3 (2.5–4.1)	3.3 (2.5–4.0)	0.1148
Serum WBC, 1,000/*μ*L	5.91 (3.36–13.18)	7.05 (3.92–13.7)	7.005 (4.59–11.05)	0.4014
ESR	30 (5–80)	40 (2–75)	30 (2–80)	0.7183

Values are expressed as median and range.
